# A self-assembled cylindrical platform for Plk4-induced centriole biogenesis

**DOI:** 10.1098/rsob.200102

**Published:** 2020-08-19

**Authors:** Kyung S. Lee, Jung-Eun Park, Jong Il Ahn, Zhuang Wei, Liang Zhang

**Affiliations:** Laboratory of Metabolism, National Cancer Institute, National Institutes of Health, Bethesda, MD 20892, USA

**Keywords:** centrosome, pericentriolar materials, centriole biogenesis, Cep63, Cep152, Plk4

## Abstract

The centrosome, a unique membraneless multiprotein organelle, plays a pivotal role in various cellular processes that are critical for promoting cell proliferation. Faulty assembly or organization of the centrosome results in abnormal cell division, which leads to various human disorders including cancer, microcephaly and ciliopathy. Recent studies have provided new insights into the stepwise self-assembly of two pericentriolar scaffold proteins, Cep63 and Cep152, into a near-micrometre-scale higher-order structure whose architectural properties could be crucial for proper execution of its biological function. The construction of the scaffold architecture appears to be centrally required for tight control of a Ser/Thr kinase called Plk4, a key regulator of centriole duplication, which occurs precisely once per cell cycle. In this review, we will discuss a new paradigm for understanding how pericentrosomal scaffolds are self-organized into a new functional entity and how, on the resulting structural platform, Plk4 undergoes physico-chemical conversion to trigger centriole biogenesis.

## 1. Introduction

### 1.1. Centrosome: a well-organized membraneless organelle

The centrosome composes a pair of microtubule (MT)-derived structures called centrioles and an amorphous mass of pericentriolar material (PCM) [1–3]. It functions as the main MT-organizing centre in animal cells and plays key roles in promoting various cellular processes, including but not limited to spindle formation, chromosome segregation and cytokinesis. Not surprisingly, aberrations in centrosome architecture and function are associated with the development of various human diseases, such as cancer, microcephaly, ciliopathy and primordial dwarfism [4–6].

The ultrastructure of centrioles was revealed by electron microscopy as early as the 1970s [7], and subsequent studies suggested that barrel-shaped centrioles do not undergo extensive structural changes during the cell cycle [8,9]. However, the way in which PCM is organized remains largely elusive, even though a network-like ultrastructure of PCM has been visualized [9,10]. This is probably because PCM is dynamically regulated throughout the cell cycle and its components are constantly turned over and replaced with those in the surrounding cytosol. Super-resolution imaging technologies, which operate beyond the diffraction limit of optical microscopy, have given us new insights into how PCM is organized. Fascinating results obtained from human and *Drosophila* cells [11–13] show that various PCM proteins are remarkably well organized, exhibiting concentric localization patterns around a centriole.

Given the dynamic turnover of their components in an open cytoplasmic environment, the mechanism in which membraneless centrosomes maintain their shape and architecture remains puzzling. One idea put forward to explain the formation of membraneless organelles is liquid–liquid phase separation (LLPS), a physico-chemical process that allows the constituent components to de-mix themselves from other cytosolic molecules and generate unique biomolecular condensates [14–16]. Indeed, studies with a *Caenorhabditis elegans* outer PCM scaffold, Spd5, show that it forms a network-like assembly through a somewhat liquid droplet-like phase-separating ability [17,18], although similar liquid-like properties so far have not been reported for its presumptive functional orthologue, *Drosophila melanogaster* Centrosomin (Cnn) [19]. Nevertheless, the network-like structure observed in Spd5 and Cnn offers a glimpse into how outer PCM is organized in these organisms. In addition, the organization of inner PCM, which is thought to be more ordered than the expanded mitotic PCM in the outer region, is beginning to emerge.

### 1.2. Centriole biogenesis and the role of inner pericentriolar material components

Centriole duplication occurs once per cell cycle. To maintain genomic stability, it is essential to tightly control this event [20,21]. In humans, centriole duplication is initiated by the concerted actions of inner PCM proteins such as Cep192 [22–24]; Cep152 [25,26]; and Plk4, a Ser/Thr kinase and conserved key regulator of centriole duplication [27,28]. A recent study with Cep152 and its binding scaffold, Cep63 [29–31], demonstrated that they cooperatively generate a heterotetrameric complex that exhibits an unparalleled ability to self-assemble into a micrometre-scale cylindrical architecture capable of recruiting Plk4 at its outer edge [32]. Upon becoming active, Plk4 induces procentriole assembly culminated with the recruitment of Sas6, a major component of the centriolar cartwheel structure [33,34]. Compelling evidence suggests that the cylindrical self-assembly functions as a *bona fide* pericentriolar higher-order structure [32] that is critically required for Plk4's dynamic relocalization from around the Cep152 scaffold to a dot-like morphology [24,35,36]. That relocalization could occur through Plk4's LLPS activity that has been proposed to occur in a catalytic activity-dependent process [37,38]. Because the adoption of Plk4's dot-like localization pattern is an obligatory step for triggering centriole biogenesis, Plk4's subcentrosomal localization and function are intricately regulated both by upstream scaffold proteins such as Cep192 and Cep152 and by subsequent events that modulate Plk4's LLPS activity.

In recent years, multiple reviews have covered several areas, from identifying components for centriole assembly to PCM organization [20,39–41]. Therefore, in light of the discovery that the novel Cep63-Cep152 self-assembly is critical for regulating Plk4 function, this review will focus on discussing: (i) the scaffold proteins instrumental for Plk4 recruitment and positioning in the subcentrosomal space, (ii) the structural features of the self-assembled Cep63-Cep152 architecture, and (iii) the physiological significance of the Cep63-Cep152 self-assembly in promoting Plk4-mediated centriole biogenesis. We hope that this review offers insights into inner PCM organization and serves as a roadmap for studying the structural and functional relationship of various PCM scaffolds in humans and other higher eukaryotic organisms. Studies show that many mutations in the inner PCM scaffolds are linked to diverse centrosome-associated human disorders [4,30,42–45]. Therefore, a deeper understanding of the architecture and function of PCM scaffolds may furnish valuable insights into the etiology of various human disorders.

## 2. A two-layered system for maneuvering Plk4 recruitment and repositioning

### 2.1. Cep192 and Cep152: two distinct scaffolds for Plk4 recruitment

The advent of super-resolution imaging technologies has shed new light on the manner in which PCM is organized in various organisms. Studies with *C. elegans* and *D. melanogaster* demonstrate the presence of distinctly arranged PCM components placed at the different regions of PCM [12,13,46]. Similarly, several human PCM proteins (CEP120, Cep192, Cep152, Cdk5Rap2, NEDD1, TUBG1, PCNT, etc.) are shown to be spatially well organized and to exhibit concentrically localized patterns around a centriole [11]. Based on the appearance of their immunostained signals, the localization morphology of these proteins has been described as a ring- or doughnut-like toroid. Additional studies have shown that at least some of the inner PCM scaffolds (e.g. Cep57, Cep63, Cep152) exhibit close to a cylinder-like morphology [47–49], suggesting that these proteins are organized into distinct layers of localized regions along the barrel-shaped centriolar axis.

**Figure 1.**
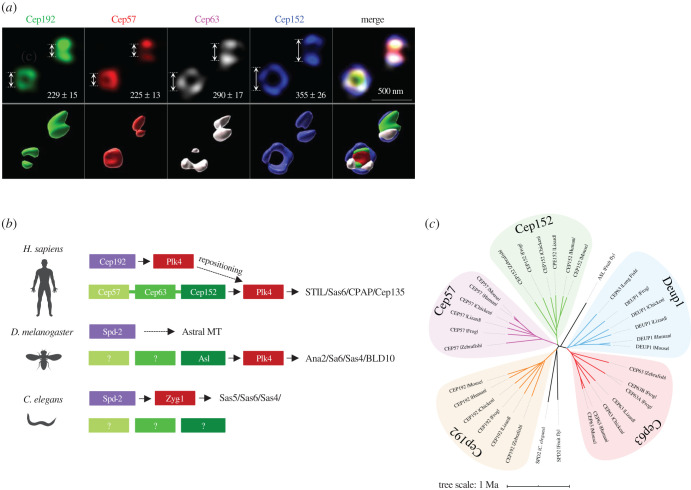
Organizational features of inner PCM scaffolds critical for the procentriole assembly pathway. (*a*) Three-dimensional structured illumination microscopy (3D SIM) analysis showing the pericentriolar localization patterns of Cep192, Cep57, Cep63 and Cep152 in U2OS cells. Cells fixed with 4% paraformaldehyde were coimmunostained with antibodies against Cep192 N-terminal, Cep57 C-terminal, Cep63 full-length and Cep152 N-terminal regions. Average diameters quantified from greater than 40 images are shown. 3D surface-rendering was carried out with Imaris v.8.4.1 (Bitplane). (*b*) Schematic diagram showing upstream scaffolds critical for recruiting Plk4/Zyg-1 in humans, flies and worms. Components are positioned relative to their locations from the centriolar axis. In *Homo sapiens*, Plk4 initially recruited to the inner Cep192 scaffold relocalizes to the outer Cep152 (repositioning) before inducing downstream events. The Cep57-Cep63-Cep152 complex is linked with a thick line. Question marks indicate no apparent orthologues found. (*c*) A phylogenetic tree for inner PCM scaffolds, including the Cep63 paralogue, Deup1, shown to be critical for deuterosome-dependent centriole amplification in multiciliated cells [31]. Note that vertebrate Cep152 and Cep192 greatly diverge from *D. melanogaster* Asl and *C. elegans* Spd-2, respectively.

Recruitment of Plk4 to the inner PCM region appears to be the first critically required step for the canonical centriole biogenesis (as opposed to centriole biogenesis dependent on non-MT fibrous bodies, called deuterosomes) under native conditions. A large body of evidence suggests that the pericentriolar scaffold proteins, Cep192 [24,50] and Cep152 [25,26], play a critical role in this event by directly interacting with the C-terminal cryptic polo-box (CPB) of Plk4 [51–53]. An early report suggested that Cep192 and Cep152 cooperate to recruit Plk4 to centrosomes and that their functions are partially redundant, because depletion of both proteins results in an additive defect in this process [50]. However, several lines of evidence suggest that they instead serve as independent physical and functional entities to promote Plk4 function. First, Cep152 localizes at a significantly outer region distinct from Cep192, which localizes near the innermost region of PCM. The Cep152 layer is at least 100 nm away from the Cep192 layer (figure 1*a*). Furthermore, unlike Cep192, whose pericentriolar localization appears to stretch up to the distal end of the centriole, Cep152 localization is confined to the proximal half of a centriole [25,50,51]. Second, Cep192 is at least partially required for normal recruitment of Cep152, but not vice versa [24,50], which means that their own recruitments are regulated in a rather hierarchical manner. One baffling observation is that the depletion of Cep192 delocalizes Plk4 from centrosomes, whereas depletion of Cep152 [50] or elimination of Cep152's Plk4-binding capacity [24] increases the level of centrosome-localized Plk4 by a few folds. Because Cep152 is abundantly present in cytosol, the depletion of the cytosolic Cep152 tether may increase the free Plk4 pool that can be recruited in excess to Cep192-loaded centrosomes [24]. Third, Cep152 forms a heterotrimeric complex with Cep57 and Cep63 [54,55], whereas Cep192 does not. Fourth, while Cep192 maintains its pericentriolar localization at all times, Cep152 is recruited in the late G1 phase to daughter centrioles (see below), which will become the new mother if duplication occurs (note that the mother centriole is already decorated with Cep152 during the previous cell cycle) [51,56]. As expected, Cep57 and Cep63 recruitment occurs almost concurrently with Cep152, although the exact timing of these events has not been sorted out.

Distinct subcentrosomal localization and function of Cep192 and Cep152 suggest that their properties could be dissimilar from each other. Indeed, at the structural level, Cep192 and Cep152 turn out to be strikingly different, though both possess a short CPB-binding motif (residues 201–260 for Cep192 and residues 1-60 for Cep152) that contains an Asp-rich sequence and an adjoining α-helical motif [51]. Analyses of primary amino acid sequences (PSIPRED; http://bioinf.cs.ucl.ac.uk/psipred/) predict that Cep192 encodes a disordered N-terminal region and an unusual β-strand-packed C-terminal domain (approx. 60% of the residues 1380–2536). On the other hand, Cep152 is predicted to generate a coiled coil-rich protein (approx. 90% of the residues 210–1300), excluding the N-terminal- and C-terminal-most regions. Notably, a coiled-coil motif of Cep152 is suggested to form a linearly arranged heterotrimeric complex with two additional coiled-coil proteins, Cep57 and Cep63 (i.e. the Cep57-Cep63-Cep152 complex as depicted in figure 1*b*) [55]. Coiled coil is well characterized as a domain that mediates protein oligomerization with mechanical strength and provides the capacity to construct higher-order structures for various biological processes [57,58].

### 2.2. Orthologues in lower eukaryotic organisms

Given the architectural conservation of centrioles from worms to humans, the components of metazoan centriole biogenesis might be conserved throughout evolution. However, in *D. melanogaster*, while the Cep152 orthologue Asterless (Asl) is demonstrably crucial for centrosomal recruitment of Plk4 [59], the Cep192 orthologue DSpd-2 is involved in an early step of PCM recruitment and astral MT nucleation [60,61] (figure 1*b*). By contrast, *C. elegans* does not seem to have an apparent Cep152 orthologue and relies on Spd-2 to recruit the Plk4 homolog Zyg-1 [62] to centrosomes [63,64] (figure 1*b*). Analyses of phylogenetic distribution of these scaffold proteins suggest that vertebrate Cep152 and Cep192 proteins substantially diverged from Asl and Spd-2, respectively, early in their evolution (figure 1*c*). In addition, Cep152-binding Cep63 and Cep57 orthologues have not been found in flies and worms (figure 1*b*), although the existence of their functional orthologues cannot be ruled out. These observations suggest that a considerable level of mechanistic variation must have occurred during evolution and that Cep152 is probably a newly added pericentriolar scaffold tailored to promote Plk4-dependent events in vertebrates.

### 2.3. Mechanism underlying Plk4's scaffold switching

**Figure 2.**
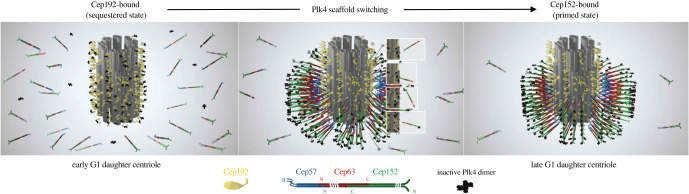
A proposed mechanism of unidirectional scaffold switching for Plk4 from the Cep192-bound state to the Cep152-bound state. In early G1, Cep192 localized around a daughter centriole recruits Plk4 at its outer end. As Cep152 (presumably as a dimeric Cep63-Cep152 complex or trimeric Cep57-Cep63-Cep152) is recruited, Cep152 snatches Plk4 away from the Cep192 scaffold (the inset diagrams) and assembles around the Cep192 layer in late G1, prompting the repositioning of Plk4 to the outer edge of the Cep152 scaffold. Note that because Cep57, Cep152 and inactivated Plk4 show a localization pattern of a ninefold radial symmetry [48,49], the Cep63-Cep152 assemblies are depicted in clusters.

Cep192 and Cep152 interact with Plk4 CPB in a competitive manner *in vivo* [24]. Analyses of X-ray co-crystal structures show that the N-terminal Cep192 (residues 201–260) and Cep152 (residues 1–60) regions bind to the same basic patch surface of CPB [51], which demonstrates their mutually exclusive nature for CPB binding. Unlike in the Plk1 polo-box domain [65], phosphorylation is not required for the interaction. Interestingly, Cep152 exhibits approximately sixfold stronger binding affinity to CPB than Cep192, thereby enabling free Cep152 to snatch away Plk4 already bound to the Cep192 scaffold in a competitive cytosolic environment [51]. Unlike Cep192, which is localized to the inner PCM region throughout the cell cycle [22,66], Cep152 (presumably in the form of the Cep63-Cep152 dimeric or Cep57-Cep63-Cep152 trimeric complex, as depicted in figure 2) is recruited to the outskirts of Cep192 in the late G1 phase [51,56], although how the timing of Cep152 recruitment is regulated remains unknown. Thus, thanks to the spatio-temporally controlled recruitment of Cep152 and its incompatible binding nature with Cep192, Plk4 molecules preloaded onto the inner Cep192 scaffold in early G1 are destined to relocalize to the outer Cep152 layer (i.e. Plk4 repositioning) as newly arriving Cep152 settles at the outskirts of the Cep192 layer (figure 2). This scaffold switching process—from the Cep192-bound state to the Cep152-bound state—appears to be required for Plk4 function because the loss of Plk4 binding to Cep152 fails to induce centriole biogenesis even though the level of centrosomal Plk4 is increased a few folds under these conditions [24,50].

Modularity is frequently observed in various biological systems, including protein–protein interactions. With that in mind, it is surprising to note that, although both an Asp-rich sequence and an α-helical motif from Cep192 or Cep152 are commonly required for CPB binding, their N-to-C binding orientations are opposite each other [51]. This is because Cep192 contains an N-terminal Asp-rich sequence followed by an α-helix, whereas Cep152 bears an N-terminal α-helix followed by an Asp-rich motif. The reversely oriented binding modes of Cep192 and Cep152 could be important because, in these situations, the N-terminal binding motif from each protein could lunge forward and grab Plk4 CPB from an opposite side, thereby avoiding steric hindrance (imagine shaking hands by approaching from the opposite direction while averting physical collision). A steric clash would not be avoided if the two proteins were to bind from the same side of Plk4 CPB. It is noteworthy that Plk4 is already repositioned at the outer edge of the Cep152 cylinder at the mother centriole during the previous cell cycle. Thus, while Cep152 recruitment is a critical step for proper Plk4 function, it is not sufficient to trigger Plk4-dependent centriole biogenesis. This means that another undiscovered event that elicits Plk4 activation may exist to induce downstream processes.

### 2.4. Physiological significance of Plk4 repositioning

Overexpression of Plk4 results in the formation of multiple procentrioles, whereas depletion of Plk4 leads to a progressive reduction in centriole numbers [27]. Furthermore, the level of intracellular Plk4 influences the ability of Plk4 to induce procentriole formation [67], suggesting that Plk4 must reach a threshold level to initiate centriole biogenesis. These results underscore the importance of tightly regulating the level of repositioned Plk4 and its subsequent activation to ensure that centriole duplication occurs precisely once per cell cycle.

So why do cells (probably all vertebrate cells containing both Cep192 and Cep152) need to reposition Plk4 from the inner Cep192 scaffold to the outer Cep152 scaffold (figure 2)? A clue may come from the fact that Plk4 must bind to the Cep152 scaffold before Plk4 initiates downstream events critical for centriole biogenesis [24,50]. A two-scaffold system may offer cells a window of time to integrate various structural and/or biochemical cues from the surrounding environment. The process of repositioning Plk4 in a three-dimensional (3D) pericentriolar space may also provide another layer of regulation to fine-tune the amount of Cep152-bound Plk4 that can be converted to the critical dot state to trigger centriole biogenesis. It is tempting to hypothesize that Cep192-dependent Plk4 recruitment functions as one step early in the process of garnering Plk4 (a sequestered state) to promptly supply a pool of Plk4 to incoming Cep152 and quickly build up the Cep152-bound Plk4 population (figure 2). Under these circumstances, recruitment of Cep152 may signal that all preceding events have been completed. The Cep152-bound Plk4 may represent a primed state that could give rise to downstream events whenever the amount of Plk4 exceeds its threshold level to sufficiently trans-autophosphorylate and generate a condensed dot state (figure 4, below). Considering that the lifespan of humans is much longer than that of *D. melanogaster* (approx. 50 days) and the average number of human cell divisions is significantly greater than that of fly cell divisions, a multistep regulatory process could be critical for tightly controlling Plk4-dependent centriole biogenesis and preserving genomic stability (an engine with more cylinders makes the power delivery smoother!). In addition, the evolutionary emergence of the coiled-coil Cep152 scaffold has probably made it possible to build a larger pericentriolar architecture that enables flies and other higher eukaryotic organisms to cope with complex biological processes.

## 3. Building a micrometre-scale cylindrical Cep63-Cep152 architecture

### 3.1. Clustering activity of Cep63 and Cep152: a driving force of self-assembly

**Figure 3.**
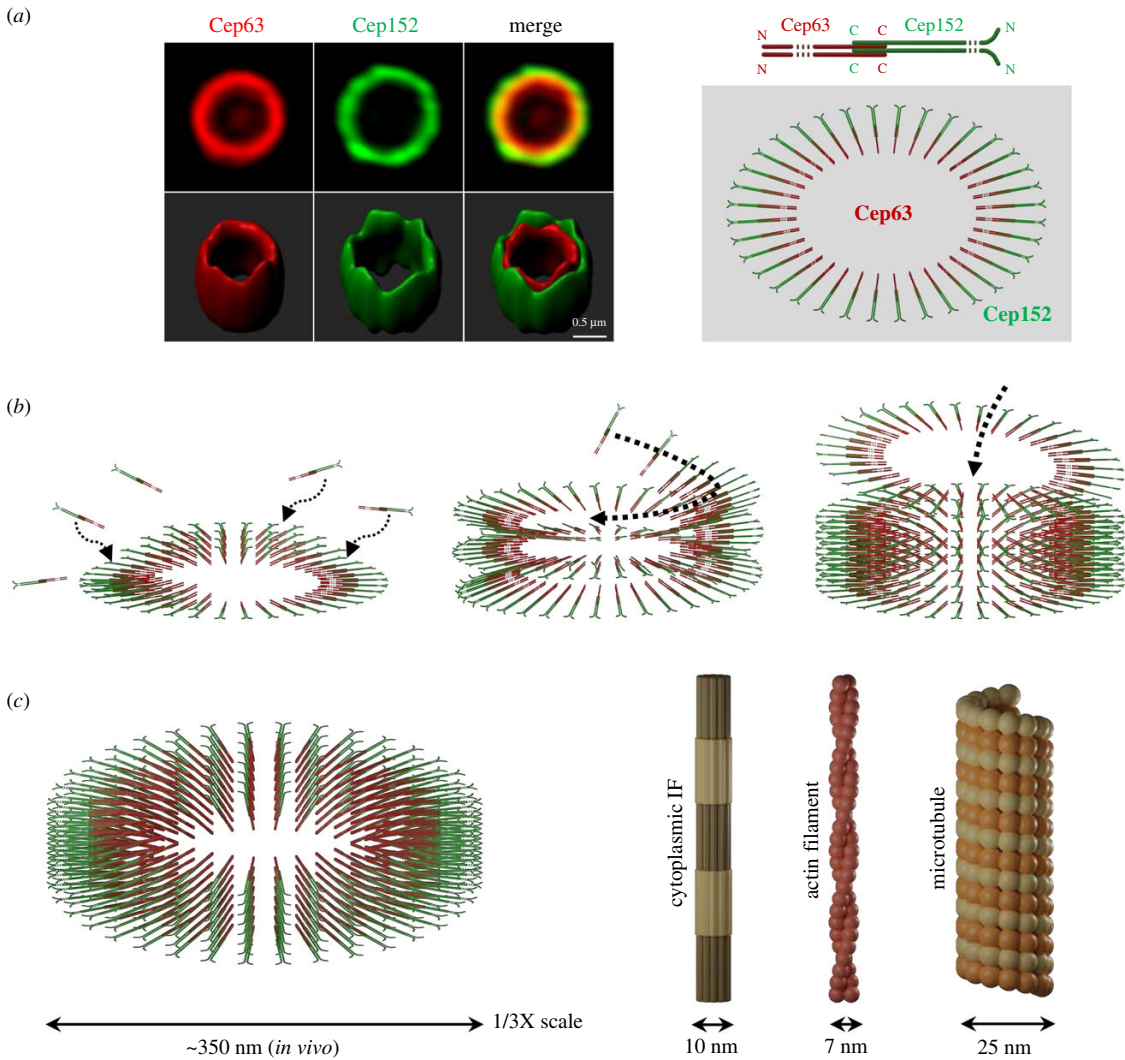
Organization of the Cep63-Cep152 self-assembly and its architectural features. (*a*) A three-dimensional SIM image and a surface rendering showing an *in vitro* self-assembled Cep63-Cep152 architecture on a two-dimensional slide glass surface. A schematic diagram depicts an antiparallelly arranged assembly in the Cep152 N-terminus-out fashion, as reported in [32]. (*b*) Hypothetical models showing three potential mechanisms that could lead to the cylindrical Cep63-Cep152 self-assembly. The third (right) stacking mechanism is disfavoured. (*c*) Schematic showing the dimension of the Cep63-Cep152 self-assembly (drawn in 0.5 X) in comparison to much-studied cytoplasmic IFs (adopted from [71]), F-actin and MT.

The first hint that Cep63 and Cep152 might cooperatively generate a higher-order self-assembly came from the striking observation that co-expression of both proteins, but not each individually, leads to the formation of hollow spherical assemblies in the cytosol of transfected mammalian cells. A similar cooperative activity was observed when purified Cep63 and Cep152 proteins were incubated in a 3D space (i.e. in a tube) under physiological buffer conditions *in vitro* (T.S. Kim, J.I. Ahn, and K.S. Lee 2020, unpublished). Biochemical and structural analyses show that Cep63 and Cep152 form a heterotetrameric complex through the side-by-side arrangement of their short (less than 70-residue-long) fragments, each containing a coiled-coil motif (approx. 40–50 residues long) and an adjoining hydrophobic residue-enriched motif [32]. Protein self-assembly is generally mediated by water-driven hydrophobic interactions because of the inherent propensity of its constituent component(s) to cluster and reach the lowest free energy state in an aqueous environment [68,69]. In line with this notion, mutations on the hydrophobic residues present in their respective motifs greatly impair the ability of Cep63 and Cep152 to generate a spherical assembly both *in vitro* and *in vivo* [70]. While these findings hint that the Cep63-Cep152 complex possesses an intrinsic physico-chemical ability to generate a self-assembly, the formation of a spherical assembly is somewhat unanticipated, given its cylinder-like localization pattern around a centriole [47–49]. Surprisingly, however, the Cep63-Cep152 complex efficiently generates a cylindrical self-assembly when placed on a two-dimensional surface [32] (figure 3*a*, left). It is possible that a component(s) or factor(s) that can substitute the surface's physical support determines the overall geometric shape of the Cep63-Cep152 self-assembly in the native pericentriolar environment.

### 3.2. Molecular basis of the Cep63-Cep152 self-assembly

Although hydrophobic motif-induced clustering is an important step in the self-assembly process, it may not account for the specific molecular recognition required to form a stable higher-order architecture. Analysis of an X-ray crystal structure covering the coiled-coil region of the Cep63-Cep152 complex reveals that the two proteins form an antiparallelly arranged heterotetramer with two parallelly aligned Cep152 helices that serve as the backbone of the four-helix bundle (i.e. the building block) [32] (figure 3*a*, right diagram). Super-resolution imaging analyses suggest that the Cep63-Cep152 self-assembly is generated by the unidirectional organization of building blocks radially arranged to form a cylindrical architecture in a Cep63 N-terminus-in and Cep152 N-terminus-out fashion (figure 3*a*). Consequently, Plk4 bound to the N-terminus of Cep152 would naturally be positioned at the outermost region of the Cep63-Cep152 self-assembly, a localization pattern analogous to that of the endogenous centrosome. Further investigation into the way the inter-building-block interactions are engaged will be critical for decoding the underlying mechanism of Cep63 and Cep152 assembly into a cylindrical architecture.

One astonishing aspect of the Cep63-Cep152 self-assembly is that a four-helix bundle just a few nanometers thick can build up the micrometre-scale architecture in a highly organized fashion. Considering the open-ended cylindrical morphology, the self-assemblies could be generated through a repeated continuous-time stochastic process that allows rod-shaped building blocks to pile up on an initial structural foundation. It is possible that the cylinders form by adding building blocks through both lateral and longitudinal interactions in a processive manner (figure 3*b*, left). Alternatively, cylinders could be formed through uninterrupted lateral additions of building blocks in an orderly spiral manner, thus resulting in a helical growth pattern (figure 3*b*, middle). These two models may not be mutually exclusive. A third possibility could be that individual ring-shaped intermediates are pre-formed before being stacked up on top of one another (figure 3*b*, right). A similar mechanism has been suggested for building stacked cartwheel structures containing *Chlamydomonas* CrSAS-6 and its Cep135 orthologue, Bld10 [72]. However, the finding that cylinders with various diameters can be formed within the same *in vitro* reconstituted sample does not support this model.

Because the diameters of cylindrical self-assemblies differ widely *in vitro* [32]*,* the way their diverse structural foundations are initially formed remains a mystery. Nevertheless, each cylindrical structure appears to maintain the same degree of curvature around the periphery of an assembly. Although the biochemical behaviour of the building block is not known, one likely explanation is that the diameter of the self-assemblies is determined early on by the local concentration of the building block and that the uniformed curvature for each assembly is achieved by the action of minimizing exposed hydrophobic surfaces in an aqueous environment. These properties are somewhat similar to the biophysical characters of amphipathic lipids, which spontaneously assemble into liposomes, micelles and membranes with various diameters. Therefore, an intriguing question would be whether Cep63 and Cep152 contain an amphipathic lipid-like primordial motif capable of driving the self-assembly process under physiological conditions.

While the cylindrical self-assembly that a heterotetrameric coiled-coil complex generates is striking, coiled coil is well documented to form other diverse structures, such as fibres, barrels, sheets and spirals [58]. The question of whether the observed self-assembling activity of Cep63 and Cep152 faithfully mimics the *in vivo* organization capacity of these proteins requires further investigation, but it is clear that the ability of a short rod-shaped Cep63-Cep152 complex to self-assemble into a micrometre-scale cylindrical architecture (figure 3*c*, left) is unprecedented. The architectural principle of building the Cep63-Cep152 self-assembly is greatly different from approximately 10 nm thick intermediate filaments (IFs) organized through both lateral association and longitudinal annealing processes of an elongated coiled-coil structure [71] (figure 3*c*, right). In addition, two extensively studied filamentous assemblies, actin filaments (F-actin; approx. 7 nm in thickness) and hollow MTs (approx. 25 nm in thickness), are made of globular proteins (figure 3*c*, right) that polymerize by harnessing the chemical energy released from the hydrolysis of ATP or GTP, respectively [73]. Therefore, the principles governing the assembly of these structures are expected to be distinct from those of assembling the Cep63-Cep152 architecture. A deeper understanding of the Cep63-Cep152 self-assembly process could provide valuable insights into the organization and function of various PCM scaffolds around a centriole. Moreover, elucidating how the self-assembly interacts with other PCM scaffolds and promotes the function of its client proteins, such as Plk4, would enhance our understanding of the physiological significance of self-assembly formation (see below).

### 3.3. Architectural properties of the Cep63-Cep152 self-assembly

The diameter of the Cep63-Cep152 cylindrical self-assembly is at least an order of magnitude larger than the three major cytoskeletal filaments (IF, F-actin and MT) (figure 3*c*), suggesting that the structural rigidity of the cylindrical self-assembly could be less than these filamentous structures, which are designed to provide enough mechanical strength to maintain cell shape and intracellular organization. Remarkably, though, Cep63-Cep152 self-assemblies turn out to be very stable and capable of maintaining their cylindrical architecture for more than 5 days *in vitro,* even under the disequilibrated conditions in which surrounding unincorporated building blocks are depleted [32]. Because a self-assembly has reached to the lowest Gibbs free-energy state through a stepwise binding process, dismantling the self-assembly would require overcoming a steep free-energy barrier. Although thermodynamically stable, the components of Cep63-Cep152 self-assemblies appear to undergo dynamic turnover with those surrounding them [32]. The dynamic nature of the assembled structure could be important for allowing other PCM proteins in the region to freely diffuse through the architecture. In addition, a wide range of diameters observed in the *in vitro–*generated Cep63-Cep152 self-assemblies suggests that the interactions between building block molecules could be flexible, thus allowing varying degrees of curvatures for the assembly. The flexible nature of the assembly is probably important for ensuring structural resilience against the various mechanochemical processes (e.g. MT assembly/disassembly and motor-driven MT sliding) that are constantly occurring at centrosomes.

### 3.4. Architectural role of Cep57

**Figure 4.**
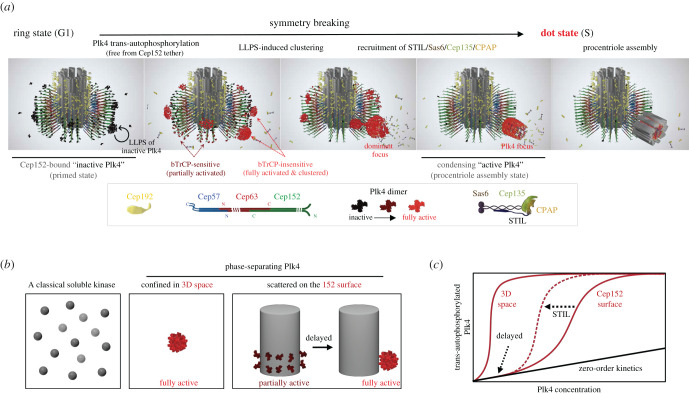
Symmetry-breaking of Plk4 on the cylindrical Cep63-Cep152 assembly, and its biochemical significance. (*a*) In G1, a fraction of Cep152-bound inactive Plk4 (i.e. ring-state Plk4) may stochastically cross a critical point of phosphorylating the CPB, thus causing the enzyme to undergo LLPS, generating patches of Plk4 condensates [38]. Based on the data obtained with the N-terminal fragment of Plk4 [37], catalytically inactive Plk4 (indicated by a black dimer) may also possess the LLPS capacity (circular arrow, first panel), which may help active Plk4 to generate condensates through a lateral inhibition self-patterning process [37,49]. A single Plk4 focus (i.e. dot-state Plk4) is thought to ultimately emerge from subsequent amplification and competition processes [49,75]. Condensed active (red, shown in cluster), but not partially active (dark burgundy), Plk4 could evade *β*TrCP-mediated suicidal degradation [38]. Incoming STIL, which activates Plk4 [76,77], can reinforce the Plk4's ring-to-dot conversion process under these circumstances. The dot-state Plk4 serves as an assembly matrix for centriole biogenesis. (*b,c*) A monomeric soluble kinase autophosphorylates its intramolecular target site (*b,* first panel) with zero-order kinetics (*c*, linear line). If Plk4 is at a sufficient concentration in a confined 3D space, its catalytic activity-dependent LLPS activity would enable it to cooperatively generate trans-autophosphorylated products as the enzyme becomes active over time (*b,* second panel), thus exhibiting sigmoidal kinetics (*c,* curved line marked ‘3D space’). Tethering Plk4 around the cylindrical Cep152 is expected to delay the early clustering process (*b,* third panel, and *c*, curved line marked ‘Cep152 surface’), providing a window of time to properly regulate Plk4's ring-to-dot relocalization while filtering out under-threshold (i.e. noise) Plk4 clusters. STIL could enhance the cooperative production of trans-autophosphorylated Plk4 and subsequent condensation by activating Plk4, thus augmenting the sigmoidal reaction kinetics (*c*, dotted arrow).

The diameter of pericentriole-localized Cep57 signals in figure 1*a* hints that they localize at or near the outer edge of the centriolar MTs. Consistent with this notion, the C-terminal domain of Cep57 exhibits a capacity to directly bind to MTs [74], while its N-terminal region interacts with the N-terminus of Cep63 and forms a trimeric Cep57-Cep63-Cep152 complex [55] (figure 2). These findings raise a fascinating possibility that Cep57 may stabilize the Cep63-Cep152 self-assembly by directly linking it to the centriolar MTs. A recent study shows that overexpressed Cep57 can recruit Cep63 and Cep152 at its outer regions, generating concentrically organized Cep57-Cep63-Cep152 signals around the longitudinal axis of the Cep57-induced MT bundles [55]. As a result of the radial arrangement of the Cep57-Cep63-Cep152 complex, Plk4 that binds to the N-terminus of the outermost Cep152 is anticipated to be at the periphery of the trimeric Cep57-Cep63-Cep152 architecture (figure 4*a*).

While these findings offer insight into the overall organization of the Cep57-Cep63-Cep152 scaffolds, we are still at an early stage of understanding how the formation of this architecture is regulated. Notably, the Cep57-Cep63-Cep152 architecture is limited to the proximal end of a centriole [48]. Therefore, how this region of the centriole is specified and whether post-translational modification of centriolar MTs such as acetylation or glutamylation plays a role in site selection are worth investigating further. Moreover, the question of whether the formation of the Cep57-Cep63-Cep152 complex itself is regulated would be worth exploring. Further study is needed to understand the mechanism underlying the assembly of the Cep57-Cep63-Cep152 architecture.

### 3.5. A cylindrical inner pericentriolar material platform for building outer pericentriolar material?

In addition to Cep57's potential role in linking the Cep63-Cep152 self-assembly to centriolar MTs, a recent report suggests that Cep57 is required for proper centrosomal localization of a pericentriolar scaffold, pericentrin (PCNT) [78], a key component of PCM organization during mitotic expansion [39,79–81]. Interestingly, a Cep57 mutant defective at recruiting the Cep63-Cep152 complex to PCM is also shown to be impaired at recruiting PCNT [78], thus raising the possibility that the Cep63-Cep152 assembly has another function in constructing PCNT organization. Furthermore, it is suggested that Cep57 interacts with NEDD1 [82], an outer PCM component critical for recruiting γ-TuRC to centrosomes [83]. In line with these findings, the depletion of Cep63 or Cep152 results in a low but significant level of defect in recruiting γ-tubulin to centrosomes [29]. These flurries of data imply that the Cep57-Cep63-Cep152 complex may play a larger role as a platform for constructing outer PCM.

## 4. Plk4: a fluidic ‘genie’ on a cylindrical platform spawning centrioles

### 4.1. The Cep63-Cep152 platform for Plk4 functionality

Exciting recent works show that Plk4 undergoes, in a catalytic activity-dependent manner, a dramatic symmetry-breaking relocalization from a ring state around a centriole to a dot state at the procentriole assembly site [37,38,76] (figure 4*a*). Because a Plk4 LLPS mutant that is catalytically active but defective in its ring-to-dot relocalization fails to garner Sas6 to the procentriole assembly site [38], the generation of a dot-state Plk4 (i.e. clustered Plk4) is probably a prerequisite for inducing centriole biogenesis. Remarkably, loss of the Cep152-Plk4 interaction, which increases the level of centrosomal Plk4 by a few folds, is shown to be sufficient to disrupt Sas6 recruitment [24], suggesting that proper establishment of the Cep63-Cep152 self-assembly is important to not only recruit Plk4 but also foster an environment for Plk4-mediated downstream events. In contrast to the Cep152-bound Plk4, Cep192-tethered Plk4 is postulated to be in a sequestered state (figure 2).

Several features of the Cep63-Cep152 self-assembly may have been tailored to promote Plk4 functionality. Because Plk4 forms a homodimer and is activated through a trans-autophosphorylation process [84], increasing proximity among Plk4 dimers would enable the enzymes to efficiently cross-activate one another. Indeed, Plk4 interacting with the long N-terminal unstructured sequence (residues 1–217) of Cep152 may possibly offer a high degree of spatial freedom on the surface of the Cep63-Cep152 assembly that could facilitate Plk4's trans-autophosphorylation events. In addition, clustered localization of Cep152 around a centriole [48,49] could promote Plk4's cross-activation and subsequent LLPS (see below). Given that the average copy number of Plk4 per centrosome is estimated to be fewer than 100 [85], the flexible nature of Plk4-bound Cep152 could be particularly important to properly activate Plk4. Aside from this notion, Cep152 may influence the catalytic activity of Plk4 either directly or indirectly, as demonstrated for *Drosophila* Asl [86].

### 4.2. Plk4's ring-to-dot relocalization and liquid–liquid phase separation

The molecular mechanism underlying Plk4's conversion from the Cep152-bound ring-like state to a focused dot-like state has been a long-standing question. Plk4 is demonstrated to be a suicidal kinase that self-destructs through an autophosphorylated degron motif for *β*TrCP-mediated proteasomal degradation [87,88]. Therefore, to successfully assume a dot-like state and trigger downstream events, Plk4 must find a way to circumvent its own destruction. Recent studies suggest that catalytically inactive Plk4 exhibits a phase-separating activity through a flexible linker region (residues 280–305) [37,38,49], which could be, in part, the indirect consequence of stabilizing the protein by rendering the unphosphorylated form insensitive to *β*TrCP-mediated proteasomal degradation [87,88]. Furthermore, large regions between the N-terminal catalytic domain and the CPB (residues 280–550) and the CPB and the C-terminal PB3 motif (residues 790–870) are predicted to be intrinsically disordered (analysed by PONDR; http://www.pondr.com/), hinting that they may also contribute to the Plk4's phosphorylation-independent LLPS activity. However, although the catalytic activity-independent mechanism could play a role at the initial stage of Plk4 clustering [37] (figure 4*a*, first panel), the finding that inhibition of Plk4's catalytic activity annihilates its ring-to-dot conversion [38,76] indicates that Plk4 trans-autophosphorylation is centrally required for this event.

At the molecular level, autophosphorylation of the PC3 motif (at residues S698, S700, T704 and T707) within the C-terminal CPB (probably through an increased local concentration of Plk4) dramatically increases the level of exposed hydrophobic surface that causes Plk4 to cluster through LLPS [38] (figure 4*a,* red clusters). The LLPS-mediated condensate formation helps physically evade *β*TrCP recognition, thus allowing active Plk4 molecules to accumulate over time and elicit centriole biogenesis [38]. These findings suggest that autophosphorylated CPB's inherent physico-chemical activities drive Plk4's ring-to-dot conversion by efficiently coalescing activated Plk4 molecules from around a centriole (figure 4*a,* second to fourth panels) rather than from the cytosolic pool. Direct visualization of this process using time-lapse imaging with a photoconvertible recombinant Plk4 may provide a more definitive answer. Although it is not known whether Plk4 orthologues in lower eukaryotic organisms exhibit similar liquid drop-like properties, a study with *Xenopus* Plk4 demonstrates that it is capable of generating a liquid droplet–like spherical condensate in a catalytic activity-dependent manner [89].

### 4.3. Two faces of STIL: a guardian or destroyer of Plk4

While Plk4's autophosphorylation onto its CPB's PC3 motif appears to be sufficient to prompt its own ring-to-dot relocalization at its native site [38], the mechanism in which its downstream effector, STIL, contributes to this process is poorly understood. Early studies suggested that STIL recruited to the pericentriolar region selectively protects the activated dot-state Plk4 from proteasomal degradation, whereas it paradoxically promotes the autophosphorylation and degradation of the ring-state Plk4 around the region [35,90]. A subsequent study showed that the coiled-coil region (721–746) of STIL binds and protects Plk4 from degradation at the procentriole assembly site, whereas the C-terminal region (1268–1287) of STIL binds and promotes the autophosphorylation and degradation of Plk4 localized at other pericentriolar ring regions [76]. Although these data suggest that two distinct modes of STIL binding to Plk4 account for the two opposing fates of Plk4 (i.e. safeguarded versus degraded), the way in which the Plk4-STIL interactions can occur distinctively in the first place remains puzzling. Lately, molecular simulations have been used to develop mechanistic models that show how Plk4 cooperates with STIL to gain its physico-chemical capacity and drive a ring-to-dot relocalization [49,75]. Additional experimental data will be required to corroborate these models.

STIL, which binds to the C-terminal PB3 motif of Plk4 [90], is predicted to be intrinsically disordered save for its N-terminal region (http://www.pondr.com/), suggesting that it could influence the physico-chemical state of Plk4. Interestingly, Plk4 generates a dot-like focused signal in the late G1 phase even before recruiting a detectable level of STIL, and STIL potentiates Plk4's ability to generate condensates (J.E. Park and K.S. Lee 2020, unpublished), probably through its ability to activate Plk4 [77]. These findings suggest that the physico-chemical state of autophosphorylated Plk4 itself could determine the outcome of STIL binding-dependent Plk4's functionality. In this scenario, once autophosphorylated Plk4 reaches a threshold and forms a condensate, STIL binding is expected to augment Plk4's ring-to-dot conversion. If not, Plk4 is doomed to be degraded through STIL binding that promotes Plk4's suicidal activity to autophosphorylate and generate its own phosphodegron for *β*TrCP recognition. Thus, the race between phosphodegron-mediated βTrCP recognition and phospho-CPB-induced condensation could ultimately determine whether and when Plk4-mediated centriole biogenesis occurs. The molecular mechanisms underlying how autophosphorylations on these two regions are mutually regulated will require further investigation. It should be noted that a centrosomal protein, Cep85, is shown to directly interact with STIL and that this interaction is important for proper Plk4 activation and centriole duplication [91]. Conversely, the Cdk1-CyclinB complex is reported to bind STIL and prevent centriole biogenesis by inhibiting the formation of the Plk4-STIL complex [92]. These findings add further complexity to the mechanism that determines the timing of active Plk4-STIL complex generation.

Forming a stable Plk4-STIL complex is crucial for increasing the efficiency of Plk4-dependent STIL phosphorylation, which in turn promotes centriolar targeting of Sas6 [35,77,93,94] and other associated protein(s), such as CPAP [95]. Studies have shown that increasing the local concentration of Sas6 is sufficient to induce cartwheel assembly and subsequent procentriole formation [96,97]. Therefore, the spatio-temporal regulation of Plk4 from a ring state to a dot state and the formation of *β*TrCP-insensitive Plk4-STIL co-condensate is probably a crucial step to offer both physical and biochemical advantages for clustered Plk4 to efficiently trigger downstream events in a confined physical space.

### 4.4. Significance of regulating Plk4 on a pericentriolar space

Close correlation between Plk4's catalytic activity and its ability to prompt centriole biogenesis suggests that the biochemical environment could influence the process of Plk4's trans-autophosphorylation and therefore would play an important role in regulating Plk4 activation kinetics. Although Plk4 undergoes LLPS in a catalytic activity-dependent manner (see above), it is tethered to the cylindrical Cep63-Cep152 architecture prior to becoming catalytically active and dissociating from it [38] (figure 4*a*). Therefore, the mechanism underlying Plk4 activation around a cylindrical surface could be very different from a classical soluble enzyme following classical Michaelis–Menten kinetics behavior.

If an enzyme is a monomeric non-phase-separating kinase diluted in a homogeneously mixed 3D space, then its intramolecular autophosphorylation will become ratiometric, showing a linear relationship between the amount of the kinase provided and the level of the phosphorylated form produced (i.e. zero-order kinetics), as has been shown for protein kinase C [98] (figure 4*b*, dark balls; figure 4*c*, black line). However, Plk4 forms a dimer that has been shown to carry out intermolecular (probably, also inter-dimeric) trans-autophosphorylation [84]. Moreover, as Plk4 becomes active and autophosphorylates its C-terminal CPB, it gains an LLPS activity to yield condensates [38]. These unusual physico-chemical properties are expected to cooperatively generate autophosphorylated products with sigmoidal reaction kinetics (figure 4*b*,*c*).

What, then, could the significance be of positioning Plk4 around the outskirts of the cylindrical Cep63-Cep152 architecture? In a hypothetical 3D confinement, in fits and starts, a sufficient concentration of phase-separating Plk4 could rapidly generate its phosphorylated clusters that lead to an unscheduled production of the dot-like Plk4 state (figure 4*b*,*c*, marked ‘3D space’). On the other hand, if Plk4 is spatially distributed around the Cep63-Cep152 architecture via the Cep152 tether (figure 4*b*, scattered on the Cep152 surface), its ability to trans-autophosphorylate neighbouring Plk4 dimers and induce condensate formation would be kinetically slowed down. This delay in the condensation process could filter out weak, under-threshold Plk4 clusters (i.e. noises) before giving rise to a single focus of the procentriole assembly site (figure 4*b*,*c*, marked ‘Cep152 surface’). In addition, the ability of STIL to augment the condensation activity of Plk4 (J.E. Park and K.S. Lee 2020, unpublished) could help generate a steep accumulation of the trans-autophosphorylated Plk4 (figure 4*c*, dotted line), thus making the process become a toggle (on/off) switch-like mechanism that would induce Plk4's ring-to-dot localization conversion in a robust manner.

## 5. Concluding remarks

A growing body of evidence suggests that formation of a cylindrical Cep63-Cep152 architecture and regulation of Plk4 recruitment and function are inextricably linked. A holistic understanding of how these processes coordinately induce centriole biogenesis would require detailed investigations into the mechanisms underlying these events occurring in a 3D pericentriolar space. In addition to the Cep57-Cep63-Cep152 complex, a large number of PCM scaffolds are predicted to encode proteins with coiled coils (e.g. PCNT, Sas6, Cep135, Cep164, Cep250, CENPJ, OFD1, CNTRB, ODF2, CDK5RAP2, Ninein, etc.), whose homomeric activity tends to form a rod-like structure. In this regard, the self-assembling ability of the Cep63-Cep152 complex may represent only a fraction of the undiscovered self-organizing capacities of pericentriolar scaffold proteins. The observation of highly organized PCM layers further supports this view. The principles governing the self-assembly of the higher-order Cep63-Cep152 architecture may serve as a paradigm for investigating the assembly and function of other structurally related scaffolds in various organisms.

Investigations into the structure, dynamics, and mechanical properties of the Cep63-Cep152 self-assembly as well as the consequences of genetic alterations that influence the function of the architecture could generate far-reaching impacts on the frontiers of cell biology and molecular medicine. Abnormalities in the 3D Cep63-Cep152 architecture could alter the kinetics of Plk4 activation and/or the dynamics of Plk4's ring-to-dot conversion that would influence the timing and capacity of centriole biogenesis. Not surprisingly, mutations in Cep63 and Cep152 are associated with the development of human diseases, such as microcephaly and primordial dwarfism. Therefore, determining how these mutations specifically compromise the architecture and function of the Cep63-Cep152 self-assembly and dysregulate Plk4 function would help elucidate the etiology of the centrosome-associated human diseases with which they are associated [4].

## Data accessibility

This article has no additional data.
